# Commentary: A road map for future data-driven urban planning and environmental health research

**DOI:** 10.1016/j.cities.2024.105340

**Published:** 2024-12

**Authors:** Georgia M.C. Dyer, Sasha Khomenko, Deepti Adlakha, Susan Anenberg, Julianna Angelova, Martin Behnisch, Geoff Boeing, Xuan Chen, Marta Cirach, Kees de Hoogh, Ana V. Diez Roux, Manuel Esperon-Rodriguez, Benjamin Flueckiger, Antonio Gasparrini, Tamara Iungman, Haneen Khreis, Michelle C. Kondo, Pierre Masselot, Robert I. McDonald, Federica Montana, Rich Mitchell, Natalie Mueller, M. Omar Nawaz, Evelise Pereira, Enrico Pisoni, Rafael Prieto-Curiel, Nazanin Rezaei, Diego Rybski, José J. Ramasco, Rossano Schifanella, Saif Shabou, Lambed Tatah, Hannes Taubenböck, Cathryn Tonne, Daniel Velázquez-Cortés, James Woodcock, Qin Zhang, Mark Nieuwenhuijsen

**Affiliations:** a Barcelona Institute for Global Health (ISGlobal), Doctor Aiguader 88, 08003 Barcelona, Spain; b Universitat Pompeu Fabra (UPF), Doctor Aiguader 88, 08003 Barcelona, Spain; c CIBER Epidemiología y Salud Pública (CIBERESP), Melchor Fern'andez Almagro, 3-5, 28029 Madrid, Spain; d Delft University of Technology, Mekelweg 5, 2628 Delft, Netherlands; e George Washington University, Milken Institute School of Public Health, 20052, New Hampshire Avenue, Washington, District of Colombia, United States; f Florida Gulf Coast University, 10501 FGCU Blvd, Fort Myers, 33965 Florida, United States; g Leibniz Institute of Ecological Urban and Regional Development, Weberpl 1, 01217 Dresden, Germany; h University of Southern California, 90007 Los Angeles, United States; i Utrecht University, Heidelberglaan 8, 3584 Utrecht, Netherlands; j Swiss Tropical and Public Health Institute, 4123 Allschwil, Switzerland; k Drexel University, 3215 Market Street, 19104 Philadelphia, PA, United States; l Western Sydney University, Locked Bag 1797, Penrith 2751, New South Wales, Australia; m London School of Hygiene & Tropical Medicine, 15-17 Tavistock Place, WC1E 7HT, London, United Kingdom; n MRC Epidemiology Unit, Cambridge University, CB2 0AH Cambridge, United Kingdom; o USDA-Forest Service, Northern Research Station, 100 North 20th Street, Ste 205, 19103 Philadelphia, PA, United States; p The Nature Conservancy, 4245 North Fairfax Drive Arlington, 22203 Virginia, United States; q Institute of Health and Wellbeing, University of Glasgow, 90 Byres Road, Glasgow G20 0TY, United Kingdom; r European Commission, Joint Research Centre (JRC), 2749 Ispra, Italy; s Complexity Science Hub Vienna, Josefstädter Straße 39, 1080 Vienna, Austria; t University of California Santa Cruz, 1156 High Street, 95064 California, United States; u Potsdam Institute for Climate Impact Research, Telegrafenberg, 14473 Potsdam, Germany; v Instituto de Fisica Interdisciplinar and Sistemas Complejos IFISC (CSIC-UIB), 07122 Palma, Spain; w University of Turin, Via Giuseppe Verdi 8, 10124, Italy; x World Resources Institute, 10 G Street, NE Suite 800, 20002 Washington, DC, United States; y German Aerospace Centre, Linder Höhe, 51147 Köln, Germany; z Technical University of Munich Institute for Advanced Study, Lichtenbergstrasse 2a, 85748 Garching, Germany

**Keywords:** Urban and transport planning, Urban environmental health research, Urban indicators, Urban data inventory, GeoAI, Urban policy

## Abstract

Recent advances in data science and urban environmental health research utilise large-scale databases (100s–1000s of cities) to explore the complex interplay of urban characteristics such as city form and size, climate, mobility, exposure, and environmental health impacts. Cities are still hotspots of air pollution and noise, suffer urban heat island effects and lack of green space, which leads to disease and mortality burdens preventable with better knowledge. Better understanding through harmonising and analysing data in large numbers of cities is essential to identifying the most effective means of disease prevention and understanding context dependencies important for policy.

Recent advances in data science and urban environmental health research utilise large-scale databases (100s–1000s of cities) to explore the complex interplay of urban characteristics such as city form and size (Hu et al., 2021; Prieto-Curiel et al., 2023; Zhu, Qiu, Hu, Shi, Wang, & Schmitt, 2022), climate (Anderson et al., 2022; Iungman et al., 2023), mobility (Bassolas et al., 2019), exposure (Masselot et al., 2023; Rezaei & Millard-Ball, 2023), and environmental health impacts (Barboza et al., 2021; Khomenko et al., 2021; Khomenko et al., 2022). Cities are still hotspots of air pollution and noise, suffer urban heat island effects and lack of green space, which leads to disease and mortality burdens preventable with better knowledge. Better understanding through harmonising and analysing data in large numbers of cities is essential to identifying the most effective means of disease prevention and understanding context dependencies important for policy.

The Urban Burden of Disease Estimation for Policy Making project (UBDPolicy) aims to inform and strengthen evidence-informed policy and decision making for urban and transport planning in almost 1000 European cities in 31 countries (The Urban Burden of Disease Estimation for Policy Making, n.d.). By quantifying health impacts attributable to air pollution, noise, heat, lack of green space, and the wider impacts and trends associated with urban planning, the project aims to promote urban health and sustainability across Europe. While UBDPolicy focuses on European cities, it draws from related efforts at the global scale and aims to develop knowledge generation and translation approaches that are applicable outside of the European context.

A UBDPolicy workshop brought together researchers that work with large-scale urban databases, covering themes of: urban form and environment, cities and health impacts, urban form and mobility, urban forests and vegetation (Esperon-Rodriguez, Rymer, et al., 2022), and urban indicators (e.g., recreational space per capita (Cities4Forests UrbanShift, n.d.)). The discussions highlighted critical challenges and knowledge gaps in urban health research, commonly employed tools and methodologies, and novel technologies and approaches that collectively help to articulate a road map for future urban health research and subsequent evidence-informed policy. The findings are reported here.

## 1 Navigating the data maze

The challenges in large-scale urban environmental health research are multi-faceted. Data availability and quality across distinct settings and spatial scales are a principal challenge. Globally, comprehensive sociodemographic data (such as education level, ethnicity, and socioeconomic status) and urban climate data are limited, with this deficit being more pronounced in certain regions such as Africa (Prieto-Curiel et al., 2023). Limited data availability, in turn, leads to understudied regions and populations, perpetuating the inequalities that evidence-informed research seeks to mitigate. Even in regions with relatively comprehensive data collection, such as the European Union, significant data gaps exist at country-, city-, and intra-city level. Meaningful metrics at city and neighbourhood levels, such as indoor environmental exposures or intra-city variations in baseline health rates, remain somewhat unexplored. Moreover, researchers can face data accessibility issues. For example, limited access to geospatial tracking impacts researcher's ability to analyse location data and inconsistencies in data from open-source platforms — like OpenStreetMap — introduce challenges related to data processing and harmonisation across multiple locations. Adding to the complexity, the definition of urban form varies widely and can be based on distinct metrics, typically influenced by the discipline and purpose of research (Tonne et al., 2021), thus hindering consensus and data harmonisation. Diverse city definitions employed across urban centres limit comparability and meaningful cross-study comparisons. An additional challenge is the translation of insights and data (such as of the interrelation between urban mobility, transportation, and environmental health risks) into sustainable city design. This requires cross-sector collaboration, which is still not the default (Karvonen et al., 2021). Partly owed to the aforementioned challenges, there are significant areas within urban environmental health research that remain largely underexplored, such as inequalities (Cociña et al., 2022) and social and environmental justice (Davis & Ramírez-Andreotta, 2021), the influence of urban form on modal share and mobility hierarchy (Bassolas et al., 2019), intervention studies (e.g., that assess the efficacy of implementing changes to transport systems, such as parking removal and congestion charges) (Kuss & Nicholas, 2022), and determinants and motivations behind behavioural change (Avineri, 2021).

## 2 Promising solutions for complex challenges

Amid the complex challenges, there are promising solutions. Attendees of the UBDPolicy workshop have established a data inventory to enhance collaboration and harmonisation of data (Table 1). Harmonisation of disparate data sources should be driven by close collaboration between government agencies, urban planners, research institutions, open data initiatives, and other relevant stakeholders (Kumar et al., 2021). To encourage consensus and transparency in city boundary definitions, Table 2 classifies commonly employed urban boundaries in large-scale urban studies into administrative, functional, and morphological approaches. Recent advances in remote sensing hold transformative potential for understanding the constituents of sustainable urban form design and structure. These advances include the publication of different high spatial resolution layers outlining the global settlement extent (Hu et al., 2021): the World Settlement Foot-print (10 m resolution, temporal resolution: 2019) (German Aerospace Center, 2019), the World Settlement Footprint Evolution (30 m spatial resolution, temporal resolution: 1985 to 2015) (German Aerospace Center, n.d.), and the Global Urban Footprint (12 m resolution, temporal resolution: 2010–2013) (Esch et al., 2021). On such basis it is possible to gather data for all cities larger than 300,000 inhabitants across the globe (Zhu, Qiu, Hu, Shi, Wang, & Schmitt, 2022). This can permit clustering cities based on urban form or societal priorities, such as economic development and sociodemographic factors, which can contribute to a more nuanced understanding of urban dynamics and population characteristics associated with distinct city types (Taubenböck et al., 2020). Geospatial Artificial Intelligence (GeoAI) involves the integration of machine learning with geospatial data (Janowicz et al., 2020). Recent advances include traffic forecasting (Polson & Sokolov, 2017) and estimating the spatial pattern of inequalities using street images (Suel et al., 2019). GeoAI offers to advance three crucial research dimensions: spatially explicit models, problem-solving, and social sensing (Janowicz et al., 2020). The latter can aid data standardisation and “socialising the pixel” through data collection and integration from diverse sources, such as near body devices (e.g., smartwatch or smartphone) and social media, which can provide valuable insights of social dynamics, behaviours, public opinion, and patterns within the urban environment. A key challenge will be the effective translation of the vast quantities of remote sensing and GeoAI data into interpretable evidence.

## 3 The power of co-design, co-creation, and co-evaluation

Translating knowledge into impactful evidence-informed urban interventions and policy recommendations is crucial. For this, the power of co-design, co-creation, and co-evaluation should not be underestimated (Boeing et al., 2022). Engaging with participatory processes and citizen science, from refining research objectives and local data collection to policy implementation and monitoring can enhance awareness of urban environmental challenges and acceptance of change (Davis & Ramírez-Andreotta, 2021). This approach can improve local community data, allow tracking of changes and impacts resulting from local actions, and ultimately foster a greater sense of ownership. In tandem, effective monitoring and evaluation necessitates uniform, robust, and sensitive indicators. The Global Observatory of Healthy and Sustainable Cities was launched in 2022 and is an initiative that provides comparable data and indicators for assessing and scaling up healthy, sustainable, and resilient urban design and planning efforts (Global Observatory of Healthy and Sustainable Cities, n.d.). Capacity building and international collaborations are at the heart of this initiative, with a strong focus on validating policy and spatial indicators and barrier-free access to data, particularly in lower- and middle-income countries. The initiative recognises that benchmarking and monitoring cities to prioritise sustainability and health equity is required on a global scale. It supports data equity and accessibility through the use of open data and open-source tools (Global Observatory of Healthy and Sustainable Cities, n.d.).

## 4 Calls to action

**Fig. 1:**
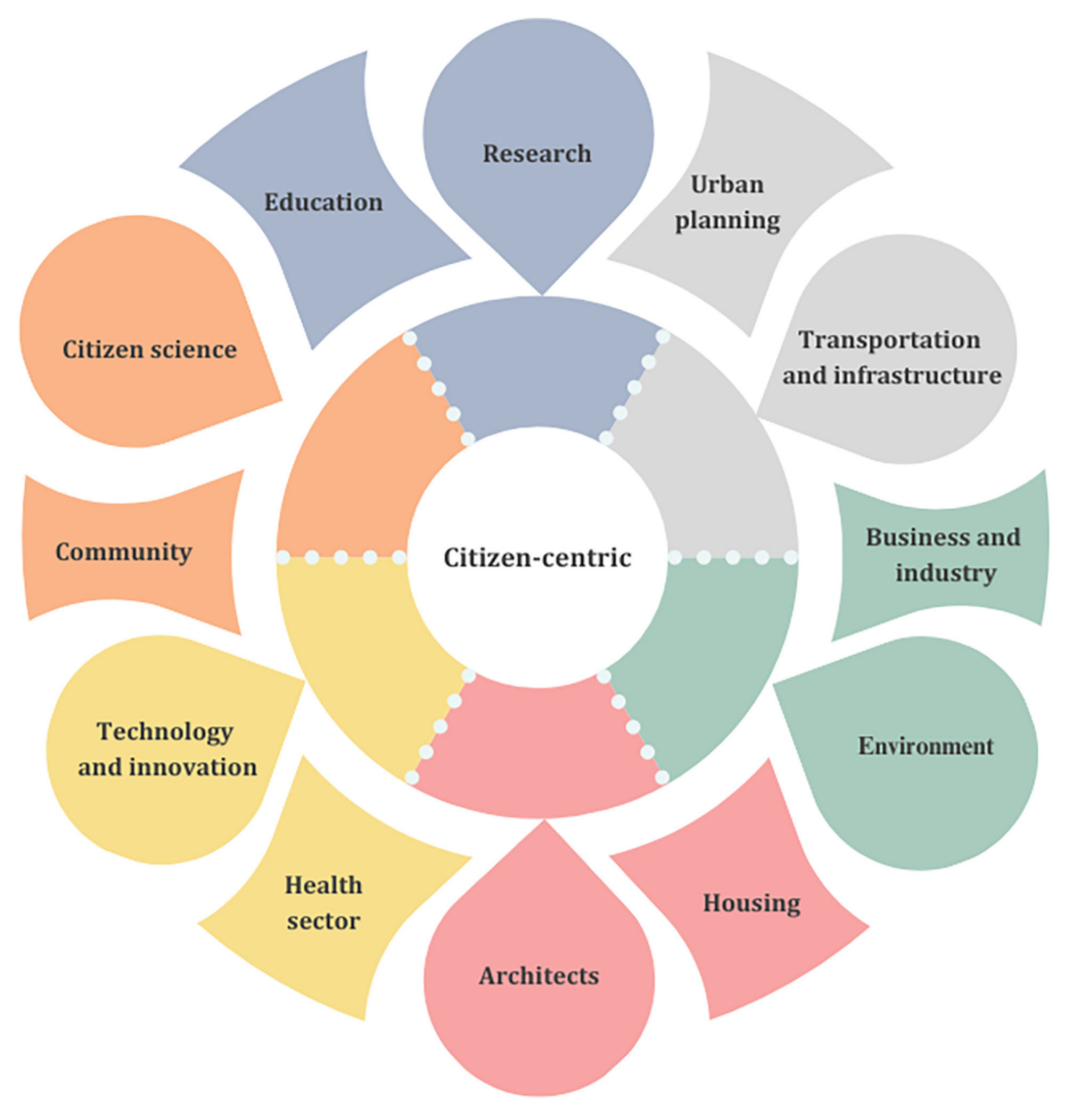
Intersectoral collaboration with a citizen- centric approach.

Addressing the diverse needs of urban populations and mitigating existent inequalities through evidence-informed policies requires collaboration across multiple sectors (Fig. 1), robust spatial data, and equity-driven practices. Enhanced efforts by local, regional, and national governments for the collection and provision of demographic and health data at finer spatial resolutions could mitigate part of the obstacles faced by urban environmental and health researchers. Citizen-oriented integration can transform current practices and drive impact (Pocock et al., 2017). This calls for context- and sociodemographic-specific research. This research should prioritise transparent and replicable methodological choices and adoption of reproducible spatial indicators for monitoring health burdens over time. Future urban health research requires greater effort for harmonised data and methodologies, collaboration, and should seek to integrate various data types at fine scales, embrace open science and data, and focus on a transdisciplinary citizen-centric approach (Panel 1 and Fig. 1). Urban environmental health researchers share a united mission of improving the health and sustainability of cities worldwide; a guide to the roadmap is here, and now it's time to implement it.

### 4.1 Panel 1: calls to action

Scientific community

- Greater data harmonisation.
- Interdisciplinary collaboration among researchers.
- Greater transparency of methodological choices and how these influence results.
- Utilisation of novel and open-source indicators and tools in pursuit of sustainable and equity driven goals and research.
- Incorporation of fine-scale data adaptable to different political units.
- Greater exploration of inequalities in large-scale urban studies; including within-city and between population subgroup analyses.

Local, regional, and national governments

- Increased efforts for uniform, open-access, and high quality local-level demographic and health data, at high spatial resolutions.
- Greater collaboration with the scientific community to share best practices and increase dialogue.
- Increased accountability across sectors, to enhance awareness of the complex relationship between urban form, environment and health.
- Encourage public citizens to provide qualitative insights of experiences and perceptions of the urban environment and proposed interventions and policies.
- Greater engagement with citizen science, ensuring underrepresented and vulnerable groups are heard.

## Figures and Tables

**Table 1:** Data inventory summary derived from the UBDPolicy workshop to enhance collaboration and harmonisation of data.

Theme	Variables/databases	Spatial resolution	Geographical regions
Population	Global Human Settlement Layer (European Commission's Joint Research Centre, n.d.)	100 m, 1 km, 3 arcsec, 30 arcsec	Global
Built environment	Global Human Footprint (Esch et al., 2021)	12 m	Global
	World Settlement Footprint (German Aerospace Center, 2019)	10 m	Global
	World Settlement Footprint Evolution (German Aerospace Center, n.d.)	30 m	Global
	Imperviousness surface (Copernicus, n.d.-a)	10 m, 100 m	European Union UK
	Residential built-up (European Commission, n.d.-a)	10 m	European Union UK
	LCZs (World Urban Database, n.d.)^a^	100 m	Global
Land use	Urban Atlas (European Environment Agency, n.d.-a)	Vectorial	European Union UK
	CORINE Land Cover (Copernicus, n.d.-b)	Vectorial Raster (100 m)	European Union UK
	UK Land Cover Map (UKCEH, n.d.)	Vectorial Raster (10 m, 25 m, 1 km)	UK
Street design and transport planning	Road network (ArcGIS, n.d.)	Vectorial	Global
	GTFS (General Transit Feed Specification, n.d.)^b^	–	Global
	DGMOVE-22 (European Commission, n.d.-b)^c^	–	European Union
Air pollution	ELAPSE (ELAPSE, n.d.)	–	European Union
	Tropomi (TROPOMI Mission Performance Center (MPC) consortium, n.d.)	Raster (7 × 3.5 km)	Global
	SHERPA (European Commission, n.d.-c)	Raster (~6 km^2^)	Europe
	Urban PM2.5 Atlas (European Commission, n.d.-d)	City (TROPOMI Mission Performance Center (MPC) consortium, n.d.)	Europe
	Urban Air Quality (Milken Institute School of Public Health, n.d.)	City	Global
Noise	Noise maps (European Environment Agency, n.d.-b)	Vectorial Raster	European Union UK
Green space	NDVI (U.S. Geological Survey, n.d.-a)	30 m	Global
	Street tree layer (Copernicus, n.d.-c)	Vectorial	Europe (urban areas)
	Tree cover (Copernicus, n.d.-d)	10 m, 20 m	Europe
	EVI (USGS, n.d.)	250 m	Global
	NatureScore (NatureQuant, n.d.)	Vectorial	Europe United States
	Tree canopy cover (NASA, n.d.)	30 m	Global
	UBD Policy Urban Forests (Esperon-Rodriguez, Tjoelker, et al., 2022)	–	Global (164 cities)
Heat/temperature	UrbClim (ECMWF Confluence, n.d.)	100 m	Europe (100 cities)
	Landsat (U.S. Geological Survey, n.d.- b)	30 m	Global
	Temperature-related mortality (Zenodo, n.d.)	City	Europe (854 cities)
	Temperature health impact projections (Lab E and HM, n.d.)	City	Europe (854 cities)
Health	Eurostat (Eurostat, n.d.)	NUTS, City, National	Europe
	Infant Mortality (CIESIN, 2000)	Raster (1 km)	Global
	Healthcare Access (Heidelberg Institute for Geoinformation Technology, n.d.)	Vector	Global
Socioeconomic	Human Development Index (SHDI) (Global Data Lab, n.d.)	Sub-national	Global
	European Social Progress Index (European Commission, n.d.-e)	Vectorial Regional level (NUTS2)	European Union
	Population at Risk of Poverty (European Commission, n.d.-f)	Vectorial Regional level (NUTS3)	Europe
	Unemployment rate (European Commission, n.d.-g)	Vectorial Regional level (NUTS2)	Europe

Abbreviations: Local Climate Zones (LCZs); General Transit Feed Specification (GTFS); Normalized Difference Vegetation Index (NDVI); Enhanced Vegetation Index (EVI); Urban Climate Model (UrbClim); Nomenclature of Territorial Units for Statistics (NUTS).

a Local Climate Zones classify urban and rural landscapes into 17 standard classes (Zhu, Qiu, Hu, Shi, Wang, Schmitt, et al., 2022).

b Public transportation timetable data and associated geographic information.

c Survey of transport modes in the EU.

**Table 2:** City definitions commonly employed in large-scale urban studies

Approach	Description	Advantages	Disadvantages	Examples of city definitions	Database	Geographical regions
Administrative	Based on administrative and political boundaries.	Boundaries are often set by a municipal or local government and therefore align with legal and political structures and resultant policies. Health data is often recorded at administrative level.	May not capture the functional relationships between different areas, leading to missed insights into the economic and social dynamics of the urban environment. The populations under study do not always align with administrative regions.	Local administrative boundaries, with ≥50,000 inhabitants	Urban Audit (European Commission, 2018)	Europe
				Administrative boundaries, with ≥100,000 residents Administrative boundaries defined in the Population Census	SALURBAL study (Quistberg et al., 2019)	Latin America
					National Bureau of Statistics of China (China NB of S of, 2021)	China
Functional	Based on functional relationships between different areas, that emphasises travel patterns and economic connections.	Reflects the movement of urban residents and thus provides a holistic view of urban dynamics. Functional definitions can adapt to changes in commuting patterns and economic ties over time.	Definitions can vary dependent on criteria used.	Functional Urban Areas	Urban Audit (European Commission, 2018)	Europe
				Metropolitan areas efined by population density, residential, and commercial infrastructure and population size	United States Census Bureau (United States Census Bureau, 2016)	United States
Morphological	Based on empirical data (e.g., building coordinates or polygons) that generates settlement masks.	Using satellite imagery and spatial data provides an objective and measurable definition. Can capture changes in urban form over time and accommodate urban sprawl.	Poses challenges when merging with health and socio-demographic data. May exclude areas that are functionally part of the city but not fully built-up. May not capture social or economic ties that define a city.	Continuously built-up areas, with <200 m between two buildings and ≥10,000 inhabitants	Africapolis (OECD/SWAC, 2018)	Africa
				Urban Morphological Zones defined as a set of urban areas laying <200 m apart (European Environment Agency, 2006)	CORINE land cover (Copernicus, 2012)	Europe
				Categorised into three tertiles of low, moderate, or high based on built environment characteristics (Nguyen et al., 2019)	United States Census Bureau (United States Census Bureau, 2016)	United States
				Morphological Urban Areas defined as territorially contiguous settlement area that can be distinguished from low-density peripheral and rural hinterlands (Taubenböck et al., 2019)	Remote sensing (Esch et al., 2017) OpenStreetMap (Open Street Map, 2017) Global Administrative Areas (Database of Global Administrative Areas v3.2, 2018) United Nations (United Nations Department of Economic and Social Affairs Population Department, 2014) WorldPop (Tatem, 2017)	Global
				Urban clusters, with ≥100,000 inhabitants	Global Urban Footprint Dataset (Nguyen et al., 2019)	Global
				≥1500 inhabitants per km^2^	CIESIN (Centre for International Earth Science Information Network (CIESIN), 2016)	Global
				Degree of urbanisation defined by population size, density, and contiguity of populated grid cells	Global Human Settlement Layer (Dijkstra et al., 2021)	Global

## Data Availability

No data was used for the research described in the article.
